# Lipid-based nanoparticles external triggered release strategies in cancer nanomedicine

**DOI:** 10.1186/s12951-025-03704-4

**Published:** 2025-10-10

**Authors:** Abdulaziz Alhussan, Louise Ho, Yao Zhang, Harrison Fan, Arash Momeni, Cedric Brimacombe, Pieter R. Cullis

**Affiliations:** 1 Department of Biochemistry and Molecular Biology, University of British Columbia, Vancouver, BC V6T 1Z4 Canada; 2 School of Biomedical Engineering, University of British Columbia, Vancouver, BC V6T 1Z4 Canada; 3 Polymorphic BioSciences Inc, 2665 East Mall, Vancouver, BC V6T 1Z4 Canada

**Keywords:** Triggered drug release, Cancer nanomedicine, Lipid nanoparticles, External stimuli, Chemotherapy delivery

## Abstract

Nearly half of humanity will develop cancer in their Lifetime. Current therapies, particularly chemotherapeutic drugs, face significant challenges due to the lack of tissue-specific delivery. For example, less than 0.1% of anticancer drugs administered systemically reach the tumor site, resulting in damage to healthy tissues and leading to a wide range of side effects. An effective strategy to address this problem is the encapsulation of chemotherapeutic drugs within nanoscale synthetic lipid structures, known as lipid-based nanoparticles (LBNPs). LBNPs can enhance a drug’s circulation half-life in the bloodstream and exploit the enhanced permeability and retention (EPR) effect. These delivery systems have led to the approval of more than 20 FDA-approved chemotherapeutic drugs. The greatest advantage is often improved pharmacokinetics, which enables a higher maximum tolerated dose while maintaining similar therapeutic efficacy and reducing side effects. However, a key limitation is that in many cases LBNPs are too stable, with free drug released very slowly, which limits anticancer efficacy. Consequently, externally triggered strategies have gained increasing attention, as they allow site-specific and on-demand release of LBNP contents at the tumor, thereby overcoming this stability barrier and enabling higher tumor-specific drug concentrations with fewer systemic side effects. This article reviews recent advances in externally triggered release mechanisms for LBNPs, including thermal, ultrasound, radiation, magnetic, and light-based approaches, and examines their potential integration into clinical cancer settings.

**Graphical Abstract:**

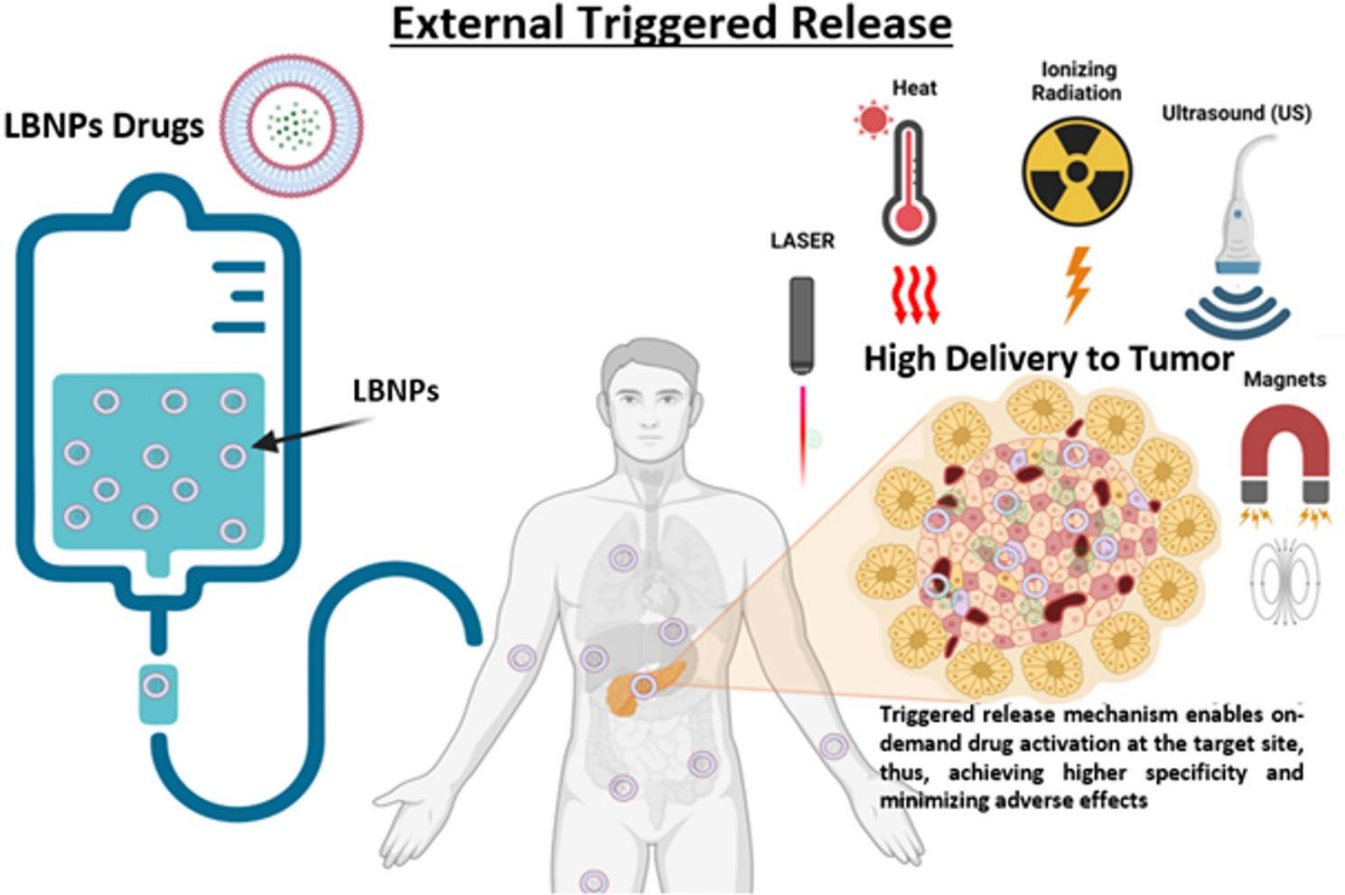

## Introduction

According to the World Health Organization (WHO), cancer is responsible for about 1 in 6 deaths globally [1]. Current estimates suggest that approximately 40% of individuals will develop cancer during their lifetime [2]. In 2020 alone, there were approximately 19.3 million new cancer cases and 10 million recorded cancer-related deaths worldwide [2]. A large proportion of cancer patients receive chemotherapy as part of their treatment regimens. Chemotherapy involves administering a small-molecule drug that interferes with cellular processes involved in active cell division, disproportionately targeting fast-dividing cells such as cancer cells [3, 4]. One main challenge in chemotherapy is that free drugs circulate throughout the body, affecting not only cancer cells but also healthy, fast-dividing cell types, which often leads to serious side effects [4, 5], while only a small fraction of the drug reaches the tumor site [6–8]. This problem is often compounded by the presence of the tumor microenvironment (TME), which reduces the access of chemotherapeutic drugs to the tumor site [5, 6].

LBNPs, including liposomes and other related structures, are established drug carriers that can improve the delivery of chemotherapies. Different techniques have been developed to rapidly generate liposomal LBNPs, efficiently load anticancer drugs into them, and extend their circulation lifetimes following intravenous administration [9–12], thereby increasing drug accumulation within tumors [12–14]. Due to their small size and prolonged circulation, LBNPs can accumulate more effectively in tumors because of the highly vascular TME and leaky blood vessels, a phenomenon known as the EPR effect [15–19]. As much as 1–5% of the injected dose per gram of tumor tissue can be found within tumors, compared to less than 0.1% of the free-form drug [20, 21]. One example is the FDA-approved drug Doxil^®^, in which doxorubicin (Dox) is encapsulated within an LBNP [22]. Although current LBNP drug systems reduce chemotherapy-associated side effects, the overall magnitude of clinical improvement is generally modest [23, 24]. This is largely due to the slow release of drugs from the liposomes and the inability to selectively release their contents within targeted tissues [12, 25, 26]. To address this limitation, researchers have increasingly turned to triggered-release systems, which release their payloads, in response to specific stimuli, within tumor tissues, thereby enhancing potency while reducing off-target effects [27–32]. These systems require both a trigger and a release mechanism. In theory, this strategy could greatly enhance the specificity of therapeutic delivery, improve anticancer efficacy, and minimize systemic side effects.

**Fig. 1:**
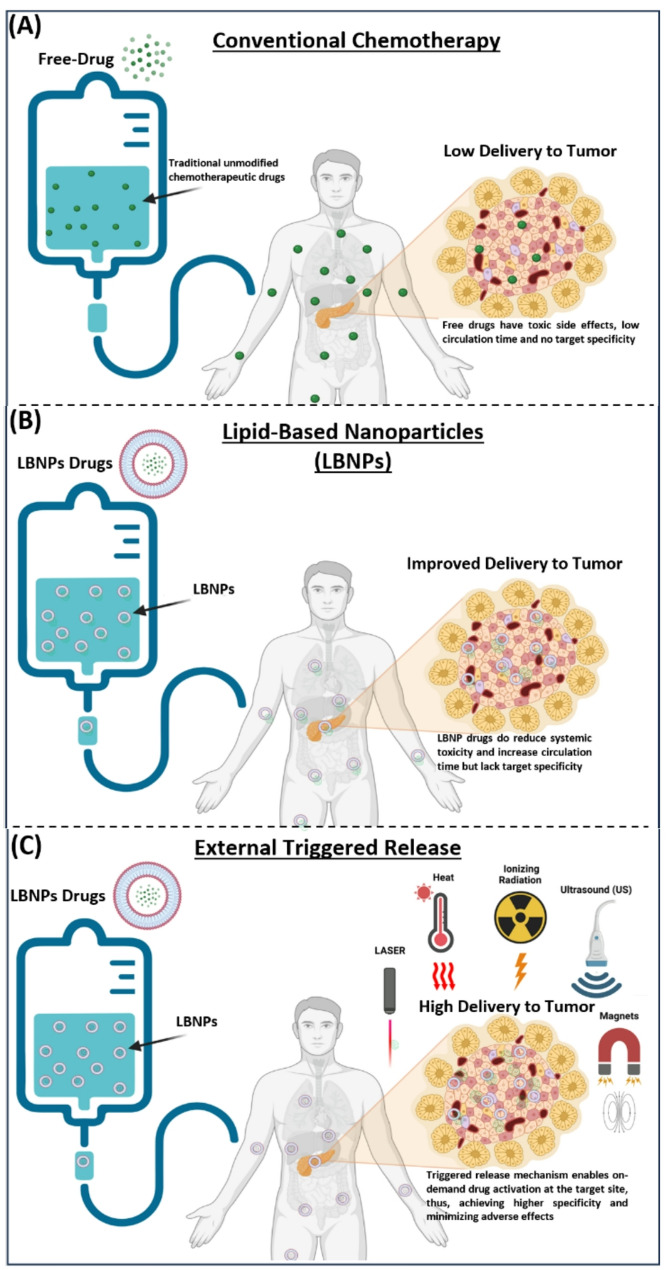
Methods for external LBNP-triggered release for drug delivery. **(A)** Free drugs (conventional chemotherapy) often have short circulation times and cause significant side effects in normal tissues. **(B)** LBNP-encapsulated drugs can improve clinical outcomes by increasing circulation time and reducing side effects but still lack the desired level of specificity for tumor tissues. **(C)** Various stimuli can be used to alter the nanoparticle structure and trigger the release of therapeutic agents from LBNPs at the tumor site, including laser, temperature control, ionizing radiation, ultrasound, and magnetic fields. This enables targeted and controlled drug activation at the desired site, thereby allowing higher specificity, reducing side effects, and enhancing overall treatment efficacy. Created with BioRender.com

Internal stimuli such as pH, enzymes, or redox gradients have been extensively investigated for this purpose; however, their effectiveness is often constrained by variability within the TME, which is highly variable between patients, tumor types, and even within different regions of the same tumor. This variability makes drug release unpredictable and very patient dependent [33]. In contrast, externally applied triggers (heat, ultrasound, radiation, magnetic fields, and light) enable on-demand drug release that is both spatially targeted to the tumor and temporally aligned with treatment schedules, offering clinicians a level of control and flexibility not achievable with internal triggers. Moreover, they are independent of both patient and tumor characteristics, making them applicable regardless of tumor heterogeneity or patient variability. These features make external strategies particularly attractive for clinical translation and form the primary focus of this review. Figure 1 illustrates several of these approaches and their advantages over conventional chemotherapy. Although many methods have been explored, each with distinct advantages and limitations [28], ThermoDox^®^ remains the only triggered-release system to reach clinical trials [34], leaving most approaches at the preclinical stage. Therefore, this review examines external strategies for triggered release of LBNP-encapsulated drugs, with emphasis on clinical applications, integration into existing treatment protocols, and future directions.

## Heat for triggered drug release

Thermal triggers leverage localized heating, often via external heating devices such as lasers, magnetic fields, ultrasound, or electric fields, to alter the nanoparticle’s structure and release the drug at precise sites [35, 36].

### Thermosensitive LBNPs:current status

Despite a strong theoretical rationale, triggered release systems have not yet reached the clinic, with ThermoDox^®^ being the only notable case to progress to Phase III trials [34, 35]. ThermoDox^®^, an LBNP formulation of Dox designed to release the drug upon mild hyperthermia (~ 41–42 °C), relies on the thermosensitive phase transition of the lipid bilayer, which increases membrane permeability and triggers drug release [34]. However, it failed in multiple trials [37–39], likely due to insufficient thermal control, excessive systemic leakage, and an overreliance on a single lipid bilayer to meet three competing demands: prolonged circulation, high drug retention, and rapid release under localized conditions [40]. This burden has proven too great for most single-membrane designs. Furthermore, given that tumor vasculature refreshes every minute, any delay in release or premature leakage into systemic circulation severely diminishes efficacy. Using a formulation similar to Doxil^®^, Amini et al. developed an LBNP system that combined 2 nm gold nanoclusters (GNCs) with Dox, which triggered drug release when exposed to a radiofrequency-electric field (RF-EF); the RF-EF generated localized heating of the GNCs, thereby disrupting the lipid bilayer and facilitating release [41]. Increased drug release was observed at higher incubation temperatures (42 °C and 55 °C), attributed to the sensitivity of the encapsulated GNCs to temperature rises following RF-EF exposure [41]. While promising, this and similar approaches remain at the preclinical stage, with no other triggered-release LBNP systems advancing to the clinic.

### Advantages and challenges of thermosensitive LBNPs

Localized hyperthermia for triggered release is attractive because it can work synergistically with chemotherapy, minimizing damage to healthy tissues and making it particularly advantageous for treating tumors in sensitive organs [42]. Additionally, heat generation can be achieved using a variety of energy sources capable of reaching and conforming to the tumor while minimizing damage to surrounding healthy tissues. However, despite its initial promise, thermosensitive LBNPs such as ThermoDox^®^ have ultimately failed in clinical trials. This failure underscores the difficulty of designing a single bilayer membrane that can both retain the drug securely and respond effectively to external stimuli. The ThermoDox^®^ formulation relied on thermosensitive lipids in the outer bilayer to trigger drug release via a phase change upon heating [40]. However, incorporating these lipids compromised the stability typically provided by conventional liposomal formulations, which use DSPC and cholesterol to create a rigid, stable bilayer. Another disadvantage is the challenge of achieving uniform and precise temperature control within heterogeneous tumor tissues, as overheating can damage nearby healthy structures while insufficient heating may fail to trigger effective drug release.

## Ultrasound: a non-invasive triggered release modality

**Fig. 2:**
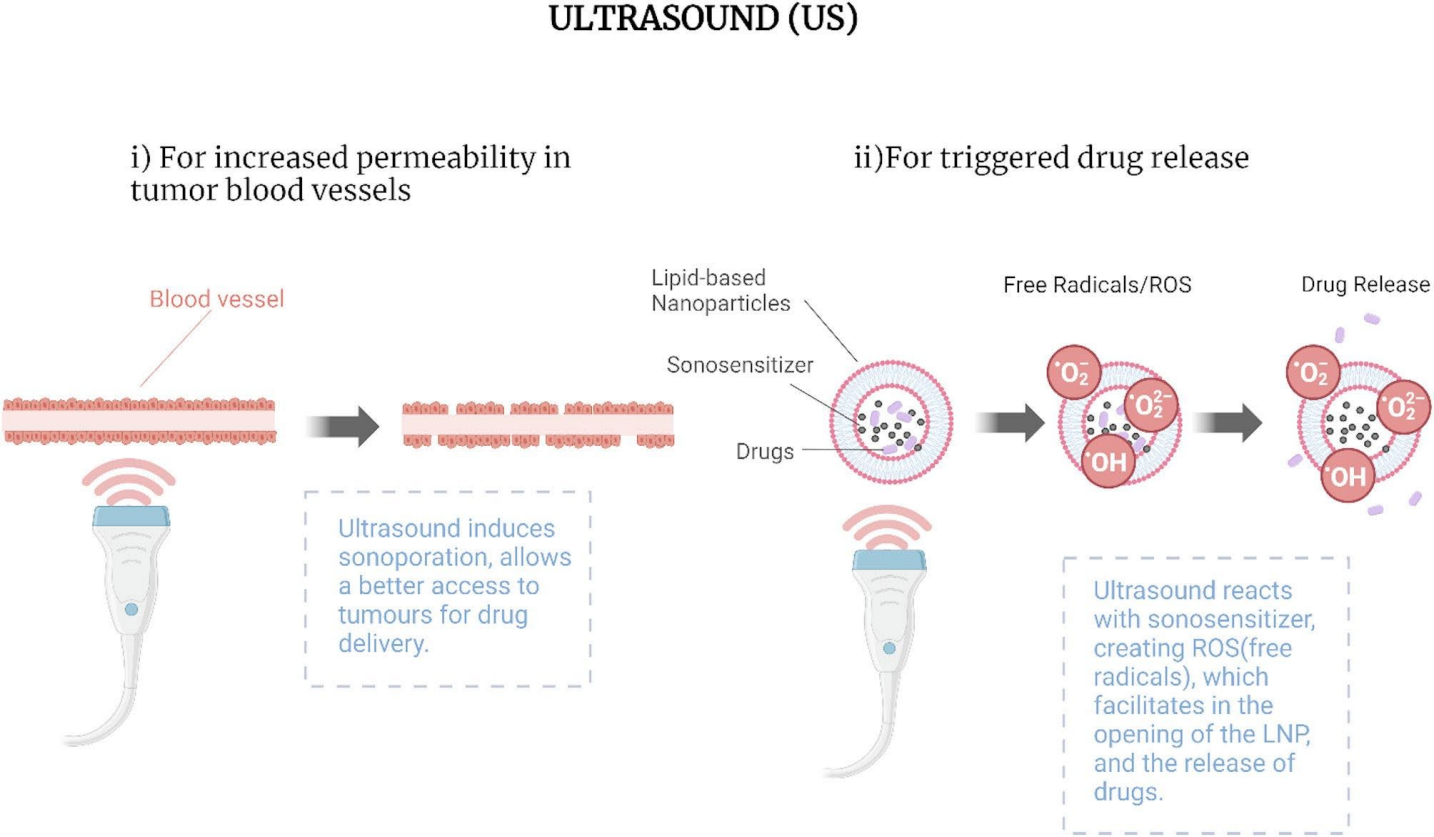
Synergistic effects of US-triggered LBNP-mediated drug delivery. **(i)** US induces sonoporation, temporarily disrupting the blood vessel barrier to enhance tumor access for drug delivery. **(ii)** US interacts with a sonosensitizer to generate ROS, which facilitates LBNP opening and the release of encapsulated drugs. Created with BioRender.com

A promising mechanism for triggered release is the use of acoustic waves or ultrasound (US). US utilizes the energy from non-invasive sound waves with frequencies above the upper audible limit of human hearing to induce mechanical vibrations or heat within the drug carrier, causing it to release the encapsulated drug [43, 44]. US is already widely used in clinical settings for a range of diagnostic, therapeutic, and theranostic applications. It can increase the permeability of the vascular endothelium, enabling more effective delivery of LBNPs directly to tumors, such as in cases where it temporarily opens the blood-brain barrier (Fig. 2i) [45].

### Low-intensity US mechanisms for triggered drug release

Most clinical applications of US-triggered LBNP drug release to date have relied on thermal effects (i.e., localized hyperthermia), particularly with thermosensitive LBNPs such as ThermoDox^®^ [46]. However, low-intensity (< 10 W/cm²) and low-frequency (< 1 MHz) US can potentially trigger drug release through non-thermal mechanisms within a few minutes, offering a safer and more controlled alternative [44, 47–54]. The exact mechanism by which US releases drugs from LBNPs remains unclear, but it is thought to involve a combination of local mechanical pressure from the sound waves and the generation of reactive oxygen species (ROS) [44, 50–59]. Metallic nanoparticles such as iron and gold (GNPs) have been shown to increase ROS production, including hydrogen peroxide (H₂O₂), superoxide, hydroxyl radicals, and singlet oxygen (¹O₂), in vitro when combined with US [57, 60–64]. These ROS alter the permeability of the LBNP lipid bilayer (Fig. 2**ii**) [56, 57, 63, 64], enabling controlled release of the encapsulated drug at the target site. Another contributing factor is acoustic cavitation, in which bubbles in the medium oscillate and collapse, generating intense mechanical stress and localized heating that facilitate drug release. The collapse of these bubbles can create transient pores in the liposomal bilayer, allowing drugs to escape [50–54, 65]. 

### Gas-enhanced US-triggered drug release

 Another approach involves the use of microbubbles (MBs) or co-encapsulation of gas and drugs within LBNPs, with US acting as the trigger for drug release [52–54, 59, 66, 67]. In this method, gas serves both as an acoustic contrast agent for imaging and as a US-triggered release agent, enabling controlled and localized drug delivery [66, 67]. While MBs have shown promising in vivo results for enhancing targeted drug delivery, their size prevents them from penetrating the TME directly. Instead, they function by undergoing US-triggered cavitation, which temporarily increases vascular permeability and facilitates localized drug release [52–54, 59, 66]. A more effective strategy uses LBNPs (~ 180 nm, composed of DPPC/cholesterol/DSPE-PEG2000) encapsulating perfluorocarbon (PFC) nanodroplets that can be triggered by high-intensity focused US (HIFU) to release their contents via mechanical disruption [54]. US peak negative pressure induces acoustic cavitation, which superheats the liquid perfluoropentane (PFP) nanodroplets, causing a rapid liquid-to-gas phase transition. The resulting bubbles rupture the liposomal bilayer, releasing the drug within fractions of a second after exposure [54, 67]. A recent study demonstrates the therapeutic potential of US-sensitive liposomal Dox combined with MBs and a convex acoustic lens-attached US (LENS) system in melanoma treatment [68]. The integration of US and MB cavitation with liposomal carriers significantly enhanced tumor-specific drug accumulation, penetration, and retention compared to Doxil^®^. Contrast-enhanced US imaging (CEUS) enabled real-time, non-invasive monitoring of drug delivery, while focused US exposure promoted MB oscillation and cavitation, mechanically disrupting vascular and liposomal barriers to improve release precision [68]. 

### Advantages and challenges of US-triggered release

 US-triggered release offers several advantages over other methods. It is non-invasive, has deep tissue penetration capability (~ 15–20 cm for lower MHz frequencies), and provides dual functionality for both imaging and therapy. It can also precisely control where and when the drug is released, thereby minimizing side effects to normal tissues [44, 69, 70]. As US devices have become more user-friendly, compact, portable, reliable, and affordable, their availability in developing countries has increased significantly. This accessibility is particularly valuable in remote and rural areas with limited advanced medical facilities. Despite promising preclinical results as a non-thermal triggering modality, no clinical trials have yet validated US-triggered release effectiveness in humans. A major challenge is the limited understanding of its mechanisms, as results can vary widely between experiments [44]. Moreover, clinical translation requires more than successful animal studies. It demands scalable manufacturing, formulations with long-term stability under various conditions, reproducible drug loading, and regulatory approval. It is worth mentioning that both HIFU and certain low-intensity pulsed US regimens can induce immunogenic cell death (ICD) by releasing tumor-associated antigens, danger-associated molecular patterns (DAMPs), and pro-inflammatory cytokines [71, 72]. These effects can enhance dendritic cell activation and cytotoxic T-cell responses, potentially synergizing with immunotherapies. However, certain US, such as pulsed focused US, may shift the TME toward an immunosuppressive state, depending on exposure parameters and tumor type [73]. Such immune-modulatory consequences must be considered when designing US-triggered LBNP systems, particularly in combination treatment regimens. 

## Rationale and potential of ionizing radiation for triggered release

 Ionizing radiation is radiation with sufficient energy to ionize atoms by exciting tightly bound electrons (wavelengths < 100 nm, energy > 12 eV). Radiotherapy (RT) is a widely used treatment for various types of cancer that utilizes the power of ionizing radiation to penetrate through the skin and into deeper tissues, focusing on the cancerous area [74]. It is estimated that more than 50% of current cancer patients receive RT during their treatment regimen [75]. The most common type of RT treatment involves the use of external beams, which can be either photon beams (X-rays or gamma rays) or particle beams (electrons or protons) [76, 77]. Because these beam types differ in penetration depth (from tens of µm for α-particles to the ability to penetrate the entire body for MV photons) and energy deposition profiles, they are selected based on tumor location [76, 77]. Given these unique properties, the use of ionizing radiation has been investigated as a trigger for the release of drugs from LBNPs, particularly in chemoradiotherapy applications [27]. 

### Photon beams and advances in radiation-responsive LBNPs

**Fig. 3:**
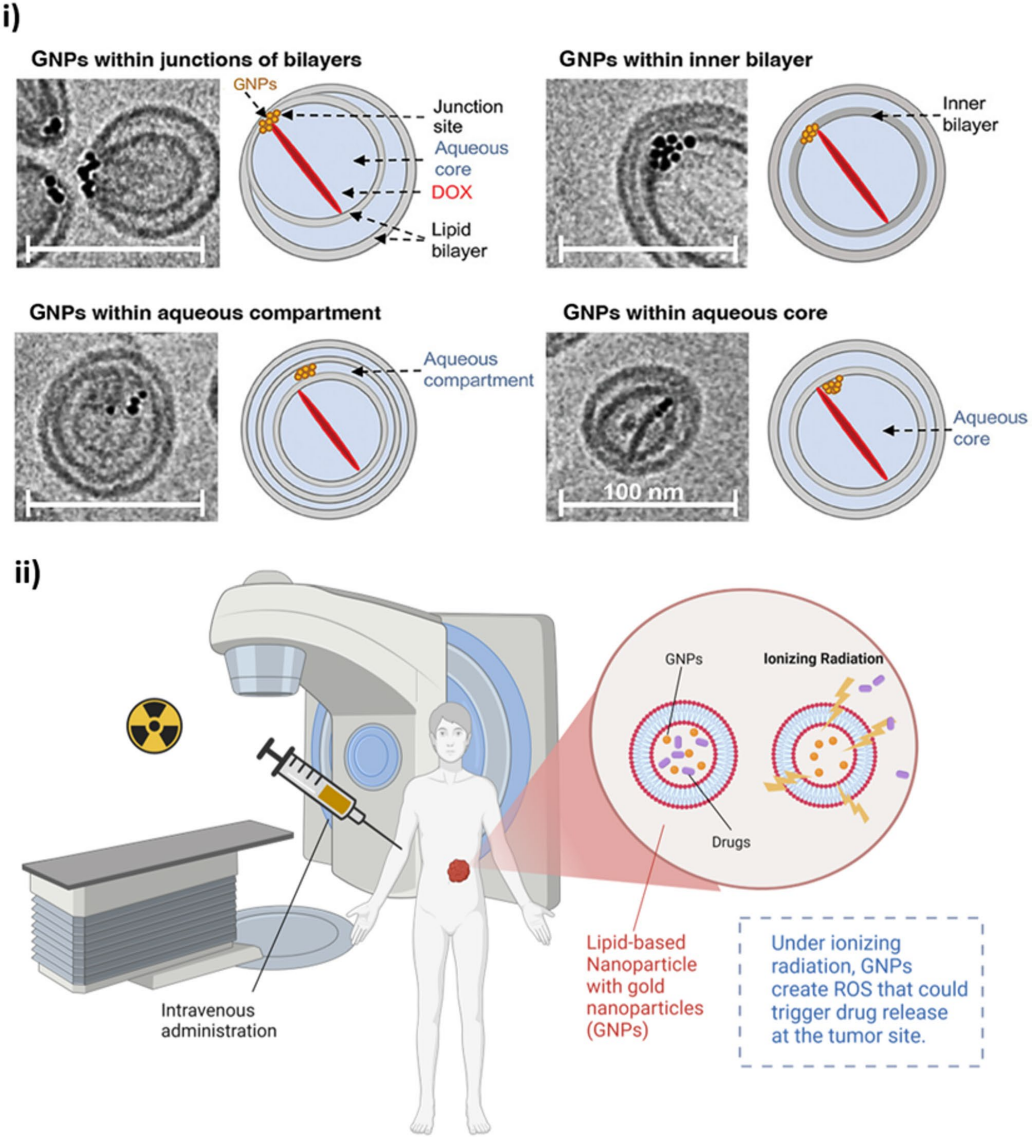
Ionizing radiation-triggered LBNP-drug delivery system for enhanced cancer therapy. **(i)** Localization of GNPs within LBNP structures containing Dox, as observed through cryo-electron microscopy. GNPs were found at different sites: within bilayer junctions, the inner bilayer, the aqueous compartment, or the aqueous core, with corresponding illustrations showing the spatial relationship of GNPs to the LBNP internal structure. The top-left image, showing GNPs within the junction of bilayers, represents the most characteristic example. Reproduced from [87], published in *Small* under the CC BY License, 2023. **(ii)** LBNPs containing GNPs are administered intravenously. Upon exposure to ionizing radiation, the GNPs generate ROS, facilitating the opening of the LBNPs and triggering the release of encapsulated drugs at the tumor site. Created with BioRender.com

 Most research in this field has focused on photon beams as the release trigger rather than particle beams [78–82]. One strategy for triggered release using conventional RT photon beams involves encapsulating GNPs in LBNPs containing Dox. When exposed to ionizing radiation, GNPs can generate ROS, which serve as a trigger for drug release [83–86]. GNPs have been successfully encapsulated within LBNPs both with and without Dox (Fig. 3i) [87]. Bromma et al. demonstrated a significant increase in GNP uptake by cancer cells in vitro when GNPs were encapsulated in LBNPs [74]. By encapsulating both Dox and GNPs within LBNPs, this approach can leverage ionizing radiation-induced ROS to facilitate the triggered release of Dox, potentially enhancing therapeutic efficacy while minimizing systemic toxicity (Fig. 3i**i**). In one example, a 6 MV photon beam (1–4 Gy) triggered the release of Dox from liposomes co-encapsulated with GNPs, verteporfin (VP; a photosensitizer), and an antisense oligonucleotide [82]. Following irradiation, VP generated singlet oxygen (¹O₂), destabilizing the liposome membrane and releasing its contents, enabling controlled, localized drug delivery [82]. This system demonstrated successful gene silencing in vitro and enhanced tumor control in vivo, showing potential for combined gene and drug therapy in conjunction with RT [82]. 

### Other emerging ionizing radiation-triggered release strategies

Aside from conventional photon beam RT, other forms of ionizing radiation have been minimally explored for drug-triggered release from LBNPs, with the notable exception of α particles in boron neutron capture therapy (BNCT). BNCT (¹⁰B(n,α)⁷Li) is a targeted cancer therapy that relies on the nuclear reaction between boron-10 (¹⁰B) and low-energy (thermal) neutrons. Upon absorbing a neutron, ¹⁰B undergoes a reaction that produces a positively charged α particle and lithium-7 (⁷Li) [88]. α particles have high linear energy transfer (LET), depositing a large amount of energy over a short distance in tissue (typically < 100 μm), making them highly destructive to nearby structures [76, 77]. Liu et al. demonstrated the use of BNCT to trigger Dox release from liposomes incorporating boron-enriched carborane into the liposomal membrane. Following neutron irradiation (~ 2 Gy), both in vitro and in vivo studies showed promising results [88]. After passive accumulation in tumors (via PEGylation for extended circulation and the EPR effect for tumor targeting), the liposomes were exposed to thermal neutrons. The resulting α particles and ⁷Li nuclei deposited energy locally, damaging cancer cells while simultaneously disrupting the boron-rich liposomal membrane and releasing Dox directly at the tumor site [88]. Despite its promise, BNCT has faced barriers to widespread clinical adoption due to challenges in boron delivery, limited neutron source availability, and complexities in treatment planning.

### Advantages and challenges of ionizing radiation-triggered drug release

Ionizing radiation-triggered drug release offers several potential advantages for cancer therapy. It enables precise, spatially controlled drug release with deep tissue penetration, while leveraging the widespread availability of radiation therapy infrastructure in cancer centers. Trained clinical teams, existing imaging capabilities for treatment guidance, and the routine use of RT in combination with chemotherapy make integration of RT-responsive LBNPs into clinical workflows feasible. Additionally, this approach may provide synergistic benefits by combining the cytotoxic effects of ionizing radiation with localized chemotherapy delivery, potentially enhancing overall therapeutic efficacy. However, several challenges remain. The primary limitation is the lack of a robust, reproducible, and rapid release mechanism at clinically relevant radiation doses. Most LBNPs do not inherently respond to standard RT beams, necessitating specialized nanoparticle engineering. Furthermore, the higher doses sometimes required to induce drug release may cause collateral damage to healthy tissues, restricting applicability to settings where RT is already indicated. As a result, this strategy remains primarily confined to oncology applications, where the use of ionizing radiation is already standard. Additionally, RT can modulate the TME by inducing ICD through mechanisms such as calreticulin exposure, ATP release, and HMGB1 secretion. These immune-stimulatory effects may provide therapeutic benefit, particularly when combined with immunotherapy [89]. However, RT can also induce immunosuppressive features within the TME, such as recruitment of immunosuppressive immune cells (e.g., M2-like TAMs) and activation of signaling pathways that favor immune resistance, particularly under specific radiation regimens and contexts [90, 91]. The balance between these opposing effects depends on factors such as dose, fractionation, and tumor type, and should be carefully considered when integrating ionizing radiation-triggered LBNP systems into clinical protocols.

## Potential of magnetic fields for triggered drug release

**Fig. 4:**
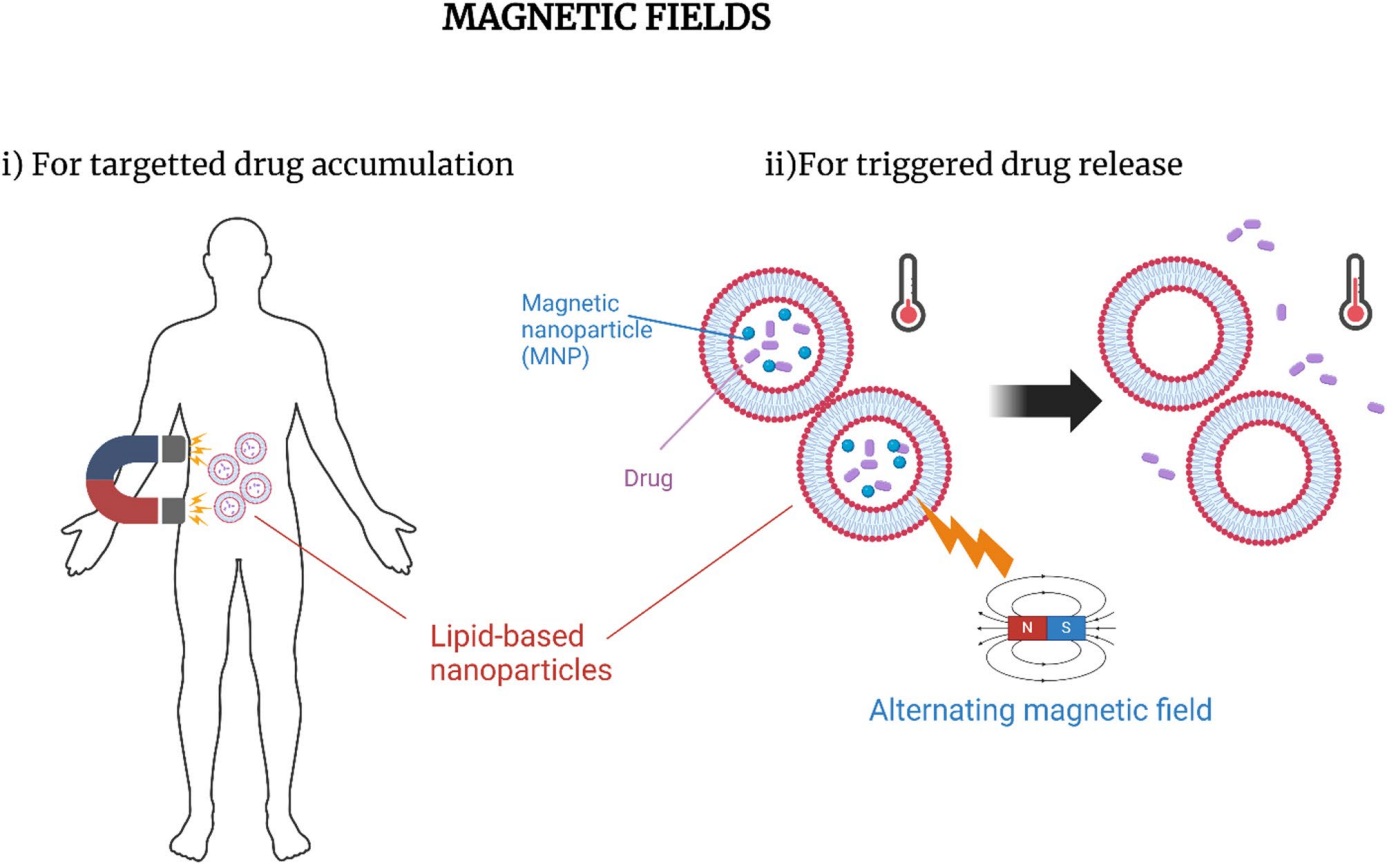
Magnetic fields for triggered-release LBNP-mediated drug delivery. MNPs are incorporated into LBNPs and administered intravenously. An external magnetic field is applied both to **(i)** accumulate the particles at the tumor location via magnetic guidance and **(ii)** generate localized heat, triggering the release of encapsulated drugs. This dual approach enhances delivery precision while minimizing systemic toxicity. Created with BioRender.com

Magnetic fields are regions where magnetic forces, generated by moving electric charges or aligned magnetic moments, exert influence, as in electromagnets or permanent magnets [92]. Magnetic nanoparticles (MNPs), such as superparamagnetic iron oxide nanoparticles (IONPs), exhibit magnetic properties only in the presence of an external magnetic field [93]. These fields can be used to trigger drug release from LBNPs via hyperthermia or mechanical oscillations when MNPs are incorporated within the LBNP structure, or to concentrate LBNPs at the target site (Fig. 4).

### Static and alternating magnetic fields

MNPs respond to both static magnetic fields (SMFs) and alternating magnetic fields (AMFs), enabling targeted drug release [93]. SMFs with sufficiently high gradients (e.g., 10–1000 T/m) can localize drug carriers within specific regions [94–97]. AMFs, which vary in amplitude over time, can induce drug release from thermosensitive carriers [93]. When AMFs with frequencies between tens of kHz and 1 MHz and field strengths of 10–100 kA/m are applied, MNPs generate heat through magnetic hyperthermia or localized vibration [93, 97–99]. This heating arises primarily from Néel relaxation (realignment of magnetic moments with the field) and Brownian relaxation (physical rotation of particles in the surrounding medium) [100, 101]. The resulting temperature increase can disrupt the LBNP lipid bilayer, increasing permeability or causing structural breakdown to release the encapsulated drug [93, 98, 100]. Even at lower AMF frequencies, hydrophobic MNPs embedded within LBNPs can generate sufficient heat to induce the gel-to-liquid crystalline transition of the lipid membrane, thereby facilitating drug release [100]. Additionally, low-frequency AMFs (LF-AMFs) can induce mechanical oscillations in rod-shaped MNPs, disrupting lipid membranes without significant heating [102, 103].

### Localized targeting via magnetic field shaping

While electromagnetic waves are constrained by diffraction limits, preventing sub-wavelength focusing [97], magnetic fields can be spatially shaped using magnetic lens arrays. For example, strong SMFs can be configured to create a field-free region. Within this region, MNPs remain responsive to AMFs, generating localized heating and disrupting LBNPs to release their drug payload [102]. In contrast, MNPs in high-SMF zones become immobilized, preventing oscillation and drug release [104]. This gating effect allows for millimeter-scale spatial targeting, ensuring localized release while sparing surrounding tissues. The approach has also been applied to co-delivery systems in which MNPs are combined with thermosensitive liposomes loaded with Dox, enabling drug release exclusively within AMF-targeted regions [104].

### Magnetic nanochain drug delivery systems

Peiris et al. developed a nanochain system (Dox-NC) comprising a 100 nm × 30 nm structure made of three magnetic IO nanospheres connected linearly, with a single Dox-loaded liposome attached [105–108]. This configuration achieved prolonged blood circulation (~ 26 h half-life), efficient tumor accumulation via the EPR effect, and precise RF-triggered release [105, 106]. Exposure to a low-power RF field (10 kHz, 5 mT) caused the IO spheres to generate mechanical vibrations that disrupted the liposomal membrane, releasing Dox into the tumor interstitium without significant temperature rise. Compared to conventional liposomal Dox, this platform achieved superior tumor deposition (7.5-fold higher in micrometastases within 2 h), effective dosing at 10–20× lower drug concentrations (0.5 mg/kg), and enhanced penetration into poorly vascularized tumor regions [106–108]. In vivo, the nanochains combined with RF extended median survival markedly (150 days vs. 44 days for controls) and demonstrated precise release, reduced systemic toxicity, and superior therapeutic outcomes, including widespread apoptosis in both vascularized and avascular tumor regions [107].

### Advantages and challenges of magnetic field-triggered release

Magnetic field-based triggering offers multiple potential advantages. MNPs embedded within LBNPs can serve as MRI contrast agents, enabling real-time carrier tracking and precise targeting [98]. Magnetic fields are generally safe and, unlike ionizing radiation, present minimal risk to surrounding tissues. Their spatial controllability facilitates localized release, reducing systemic toxicity and allowing higher therapeutic concentrations at the tumor site. Advanced configurations, such as Halbach arrays, further improve precision [93]. Additionally, magnetic parameters, including field strength, frequency, and exposure duration, can be tuned to modulate release profiles. However, several challenges have hindered clinical translation. Magnetic field gradients decay rapidly with distance, limiting deep-tissue targeting. High MNP doses may cause cytotoxicity or oxidative stress [93, 109]. From an implementation perspective, magnetic-triggered release requires specialized, standardized equipment that is not widely available, increasing both cost and complexity. Moreover, LBNPs are not inherently responsive to magnetic fields, necessitating complex integration with high MNP loads. Developing such hybrid systems that are scalable, stable, biocompatible, and capable of predictable release remains a technical challenge. As with ionizing radiation and US triggers, no magnetic-triggered LBNP system has yet reached clinical trials, with most progress confined to academic research settings.

## Light-based strategies for triggered drug release

Light-based approaches have gained increasing attention in cancer nanomedicine due to their accessibility, non-invasive nature, and capacity for precise spatiotemporal control [29, 32]. These techniques span the ultraviolet (UV; ~100–400 nm), visible (Vis; ~400–750 nm), and near-infrared (NIR; ~750–1400 nm) spectral ranges. Light can trigger drug release through photochemical processes, such as breaking photolabile bonds, or by exciting photosensitive agents to induce thermal or photomechanical effects [110].

### Short-wavelength light

One method involves photoactivatable LBNPs (paLBNPs), which release their payload upon irradiation at specific wavelengths. Chander et al. developed paLBNPs incorporating photo-switchable phosphatidylcholine analogs, AzoPC and redAzoPC, to encapsulate Dox [111]. Upon exposure to 365 nm UV Light, azobenzene moieties in the Lipid bilayer underwent trans-to-cis isomerization, causing structural disruption and releasing up to 70% of encapsulated Dox within 24 h. Interestingly, irradiation at 660 nm deep-red light yielded a comparable release profile, suggesting multi-wavelength activation potential [111].

### Visible light

**Fig. 5:**
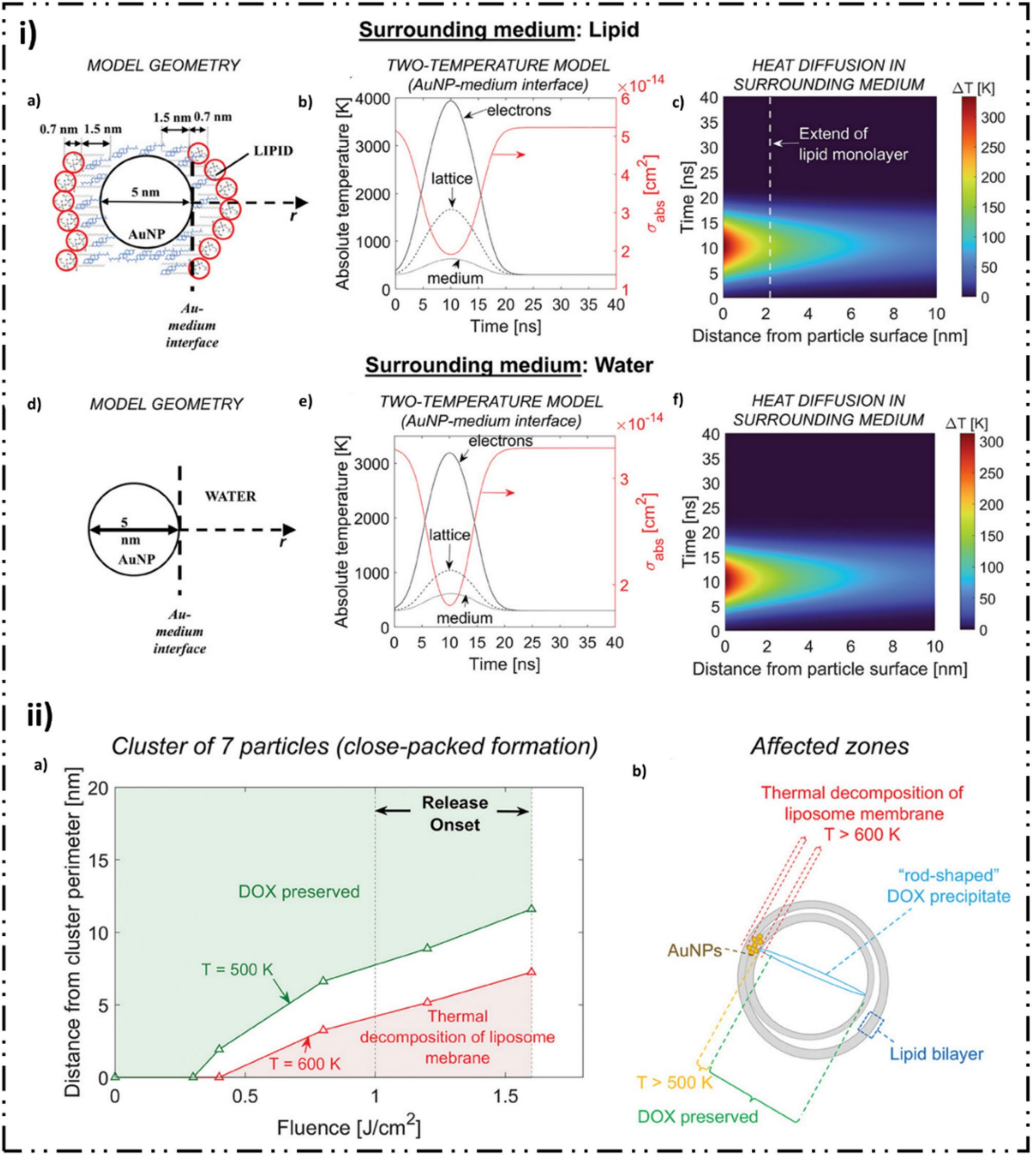
Light-based LBNP triggered release system in cancer nanomedicine. **(i)** Theoretical models and numerical simulation results examine the interaction between a single nanosecond laser pulse and AuNPs (gold nanoparticles) located either within a Lipid bilayer or in the aqueous compartments of oligolamellar vesicles. The simulations assume a laser fluence of approximately 1.6 J/cm². Panels **(a)** and **(d)** depict the geometric configurations for lipid and water environments, respectively. **(b**,** e)** The graphs illustrate absolute temperature at the interface between the AuNP and its surrounding medium (lipid or water) over time, as calculated by the two-temperature model (left axes), alongside the temporal evolution of the particle’s absorption cross-section (right axes). **(c**,** f)** The spatiotemporal profiles represent the transient temperature increase in the surrounding medium. **(ii) (a)** The zones of thermal impact around a cluster of seven particles due to collective heating are evaluated as a function of laser fluence (*F*). These zones are defined by the maximum distance from the cluster’s perimeter where a characteristic temperature is reached at the pulse’s peak. The red curve, *d(F*,* T = 600 K)*, indicates the distance where a maximum temperature of 600 K is reached, marking the threshold for liposome thermal decomposition, with the affected area represented in red. In contrast, the green curve, *d(F*,* T = 500 K)*, indicates the distance where a maximum temperature of 500 K is attained, representing the threshold for the thermal integrity of Dox. Thus, Dox stability is preserved within the green region of the plot. **(b)** A schematic illustrates the thermal impact zones under single-pulse laser irradiation, focusing on fluence values near the threshold necessary for drug release. Reproduced from [87], published in *Small* under the CC BY License, 2023

High-intensity pulsed lasers interacting with plasmonic nanoparticles, such as GNPs, can produce rapid, localized heating and photomechanical effects, including thermal expansion, thermoelastic stress, and shockwave formation, often accompanied by cavitation [112, 113]. These phenomena can transiently disrupt LBNP bilayers, enhancing delivery of otherwise membrane-impermeable agents [113–115]. One notable example is a hybrid LBNP system encapsulating Dox in the aqueous core while clustering 5 nm GNPs in internal bilayer junctions [87]. Nanosecond laser pulses (527 nm) were used to resonantly excite the plasmonic GNPs, triggering localized heating that exceeded 600 K and induced thermo-mechanical Lipid disruption and vaporization of adjacent water layers. This resulted in direct drug release into cancer cells, with an 11-fold increase in intracellular Dox concentration compared to conventional LBNPs [87]. Importantly, the rapid heating preserved Dox integrity, ensuring maintained therapeutic activity (Fig. 5).

### NIR light

NIR-triggered release often relies on energy transfer from a photosensitizer to molecular oxygen, producing highly reactive singlet oxygen (¹O₂) capable of disrupting lipid structures within ~ 100 nm LBNPs [116, 117]. Photosensitive agents, such as gold nanorods (GNRs) and indocyanine green (ICG), absorb NIR light (commonly 808 nm) and convert it into localized heat, destabilizing the lipid bilayer to release encapsulated drugs [118]. For example, Dai et al. encapsulated ICG in LBNPs to generate heat that melted the liposomal core, enabling Dox release [118]. Fang et al. employed cetuximab-functionalized hybrid Lipid-polymer nanoparticles co-loaded with irinotecan and ICG, releasing the drug upon 808 nm NIR exposure [119]. Viitala et al. embedded GNRs within LBNPs and incorporated ICG into the Lipid bilayer, achieving localized heating under 808 nm NIR irradiation to induce drug release [120].

### Advantages and challenges of light-based triggering

Light-based techniques offer some advantages due to their non-invasive nature, precise spatial and temporal control, and tunability across various wavelengths. However, despite their promise, they face challenges that hinder clinical translation. The use of UV light poses a significant challenge due to its potential to harm healthy tissues [110, 121]. Its limited tissue penetration (< 2 mm) reduces its applicability to deep-seated tumors. Therefore, researchers have focused on alternatives, such as NIR due to its lower absorption by tissues [29]. However, its application is still restricted to surface lesions due to its very short tissue penetration depth of only a few cm [121]. Techniques such as fiber optics delivery are under investigation to address this limitation, but they introduce added complexity and face regulatory challenges related to safety, standardization, and clinical implementation. Importantly, the safety profile of photosensitive agents varies significantly. For example, ICG is FDA-approved and widely used in clinical diagnostics [122], while certain gold nanostructures have demonstrated favorable biocompatibility in early-phase trials [123]. In contrast, other agents such as specific porphyrin derivatives or unmodified azobenzene-based compounds can present phototoxicity or cytotoxicity risks, necessitating molecular modifications or encapsulation strategies to improve safety before clinical use [124–127]. Additionally, incorporating photosensitive agents into LBNPs often leads to instability, aggregation, or loss of functionality over time which may pose reproducibility problems. Moreover, the high manufacturing cost and scalability challenges all resulted in no LBNP system using light as a trigger in the clinic yet.

## LBNP-triggered release considerations

### Fundamental considerations

Triggered drug release from LBNPs is controlled by several foundational factors that determine whether it can be translated effectively into the clinic. These include:

- Stability versus responsiveness: LBNPs must remain sufficiently stable during circulation to prevent premature leakage, while retaining the capacity to release their payload rapidly upon exposure to the chosen stimulus.
- Trigger accessibility: The external stimulus must reach the tumor at adequate intensity without damaging surrounding tissues, which depends on penetration depth and tissue heterogeneity.
- Release kinetics: Effective systems should match the rate of drug release with tumor perfusion dynamics, ensuring that the drug becomes bioavailable when blood flow is delivering LBNPs through the tumor vasculature.
- Spatial specificity: Ideally, release should be confined to the TME to maximize local concentrations while minimizing off-target effects.
- Biological interactions: Factors such as the TME (e.g., extracellular matrix density, immune cell infiltration) influence both penetration and payload diffusion after release.

### Clinical translation challenges

Despite promising preclinical results, no LBNP-triggered drug release strategy has yet demonstrated sufficient efficacy or reliability to achieve clinical approval [34]. Several factors hinder successful translation:

- Physiological variability and TME heterogeneity: Human tumors display significant heterogeneity in vascular permeability, pH, enzyme expression, and immune infiltration, which can limit both the penetration of triggering stimuli and the predictability of drug release [128].
- Preclinical-clinical gap: Many in vivo experiments fail to translate to humans because preclinical tumor models cannot fully reproduce the complexity of human cancers with respect to tumor heterogeneity, immune responses, and variability in stimulus penetration and drug release dynamics.
- Trigger penetration and precision: Certain triggering modalities, such as light-based systems, face inherent limitations in reaching deep-seated tumors without invasive delivery or the use of high intensities that risk collateral tissue damage.
- Formulation stability and large-scale manufacturing: Trigger-responsive LBNPs must remain stable during storage and circulation while preserving sensitivity to the stimulus, all under Good Manufacturing Practice (GMP)-compliant large-scale production [129]. Achieving this balance can be technically challenging.
- Safety and regulatory considerations: Beyond technical stability, clinical translation requires proof that LBNPs are non-leaky in healthy tissues and that the external stimuli are safe and tolerable in humans. Meeting these dual requirements introduces toxicity risks and necessitates extensive toxicological and pharmacokinetic evaluation.
- Clinical workflow integration: Some triggering modalities, particularly light-based and magnetic field-triggered approaches, demand specialized equipment, image guidance, or extended treatment times. These requirements must be compatible with hospital infrastructure and acceptable from the patient’s perspective.

Overcoming these barriers will require multidisciplinary collaboration to refine stimulus-responsive mechanisms, establish standardized operational protocols, and generate robust safety and efficacy data in human studies. These steps are essential for bridging the gap between experimental proof-of-concept and widespread clinical adoption.

### Pharmacokinetic and circulation optimization

Building on these fundamental considerations, the pharmacokinetics of LBNPs play a critical role in determining the success of externally triggered release. The four key design objectives can be summarized as follows [35]:

1. Retention: Maintain high concentrations of the drug securely within a leak-proof carrier post-administration.
2. Evasion: Avoid recognition and clearance by the reticuloendothelial system (RES).
3. Targeting: Direct the drug to the intended site effectively.
4. Release: Trigger therapeutic payload release within an optimal timeframe.

While the first two objectives are generally achievable using LBNP systems, where lipid composition determines bilayer stability and leak resistance, and PEGylation is a widely used strategy for evading the RES, the latter two objectives present considerable challenges in drug design. For external triggered release applications, LBNPs do not require active tumor targeting but instead benefit from prolonged circulation times. This is because the therapeutic effect relies on externally induced intravascular release, where drug payloads are released directly within tumor vessels during trigger application. In this approach, the spatial and temporal precision comes from the applied stimulus itself rather than ligand-receptor binding, making long systemic circulation more valuable than active targeting [6]. Additionally, active targeting of LBNPs using targeting ligands has met with very limited success [10, 15].

Once administered systemically, LBNPs circulate in the bloodstream before accumulating primarily in the liver and spleen [12]. To optimize triggered release systems, LBNP circulation half-lives should be engineered to last several hours rather than minutes, providing sufficient time for the trigger to be applied repeatedly in the region of the tumor [130]. Tumor perfusion can be estimated at approximately 0.05–0.1 mL/min/g, meaning a 100 g tumor receives about 5–10 mL of blood per minute, or roughly one full perfusion cycle every minute [131]. Therefore, increasing LBNP circulation half-life allows successive waves of drug-loaded LBNPs to continuously pass through the tumor vasculature, ensuring sustained, trigger-responsive drug release during each cycle of blood perfusion.

Drug release after triggering can occur either extravascularly or intravascularly. Extravascular release relies partly on the EPR effect but faces challenges such as limited LBNP penetration into tumors due to high interstitial fluid pressure and a dense extracellular matrix [132]. In contrast, intravascular release bypasses these issues and is therefore the preferred approach in most current research [133]. For intravascular release to be effective, triggering must coincide with peak plasma LBNP concentrations, and the drug must be fully released from its carrier within seconds of triggering. This rapid release allows the drug to move swiftly through the vasculature to the tumor site. Modeling studies suggest that pharmacokinetic release profiles are the most critical factor in achieving high localized drug concentrations in intravascular systems [134]. Understanding these circulation and perfusion dynamics provides a foundation for quantitatively comparing triggered-release LBNPs with existing chemotherapy approaches.

### Rationale and quantitative basis for triggered release

To assess the therapeutic potential of triggered-release LBNP systems compared to free Dox and conventional LBNP-Dox (e.g., Doxil^®^), we modeled tumor drug exposure based on known pharmacokinetic parameters. For a 70–80 kg adult with approximately 5,000 mL of blood, a 50 mg IV dose of Dox yields an initial plasma concentration of ~ 10 µg/mL [135]. Free Dox clears rapidly, with most eliminated from plasma within 10 min due to a half-life of 5 min, followed by clearance half-lives of 1–3 h and a terminal Phase of up to 48 h [136, 137]. Given a solid tumor perfusion rate of ~ 0.05 mL/min/g, a 100 g tumor receives about 50 µg/min of drug during the brief window when free Dox is present in circulation, totaling ~ 2.5 µg/g over 5 min. In contrast, conventional LBNP-Dox formulations circulate much longer (half-lives ≥ 20–30 h) and accumulate passively in tumors at rates of ~ 1–5% of the injected dose per 100 g tumor over 24–48 h [137, 138], providing retention in the tumor for hours due to the EPR effect. For a 50 mg dose, this translates to ~ 5–25 µg/g of cumulative exposure. A triggered-release LBNP system could greatly enhance this by synchronizing drug release with tumor perfusion, which refreshes approximately every minute. For a 100 g tumor and a 50 mg dose, if the release is triggered for 30 s of each minute (i.e., 50% of the time), and assuming conservatively that only 0.5% of the total dose is released per minute (0.25 mg/min), the local release rate would be ~ 1.25 µg/g/min. Over the course of an hour, this results in a cumulative tumor exposure of 75 µg/g, and over two hours, 150 µg/g, both of which are approximately 60× higher than free Dox and ~ 10× higher than conventional LBNP-Dox. These modeling results highlight the potential for triggered-release systems to achieve rapid, high-dose tumor exposures that are otherwise unattainable with existing drug delivery methods.

### Therapeutic gains and clinical trade-offs

While this triggering duration May seem challenging from a clinical perspective, particularly in terms of patient comfort and logistics, it enables a fundamentally different delivery paradigm. Instead of waiting for inefficient intracellular release over several days, the drug can be Liberated in real time at the tumor site, synchronized with each perfusion cycle. This approach can result in tumor drug exposures 10× higher than those achieved with conventional LBNPs and over 60× higher than with free Dox, within just one to two hours. Such a system could also increase the maximum tolerated doses (MTDs) beyond the current Dox MTD of ~ 60 mg/m² for both free Dox (limited by cardiotoxicity) and Doxil^®^ (limited by skin and mucosal toxicity) [138, 139]. This improvement is achieved by enhancing the spatial specificity of drug release, concentrating therapeutic exposure at the tumor site while minimizing off-target toxicity. It is important to note that in many clinical contexts, particularly after surgical resection of a primary tumor, general chemotherapy is administered when metastasis is evident or presumed [140]. However, the precise locations of all metastatic lesions are not always identifiable. This limitation means that externally triggered release systems, while offering localized delivery advantages, may not be applicable for whole-body chemotherapy in cases where metastatic sites cannot be accurately mapped. The clinical burden of an image-guided, outpatient-based triggered release procedure would likely be outweighed by the therapeutic benefit, particularly for aggressive tumors or in settings where rapid dose intensification is critical.

### Comparative summary of externally triggered release modalities

Table 1 provides a comparative overview of key parameters across externally triggered drug release strategies, as reported in the literature reviewed above. In the absence of external stimuli, most well-formulated LBNPs exhibit minimal drug release (< 10% over 24 h), reflecting high baseline stability. Effective systems must achieve a balance between rapid, stimulus-responsive release and maintaining sufficient circulation half-life to enable tumor accumulation. Although circulation half-life is not dictated by the triggering stimulus itself, it critically determines the therapeutic window, i.e., longer circulation provides more opportunities for external triggers to be applied effectively at the tumor site. Importantly, faster release kinetics, such as those observed with US or light-based techniques, do not necessarily correlate with clinical readiness, as demonstrated by the limited translation of these approaches beyond early-stage trials. Furthermore, there is often a trade-off between technical complexity and therapeutic precision. For example, light-triggered systems can offer highly localized spatial control but are limited by tissue penetration depth and reliance on specialized equipment. In contrast, ionizing radiation- and US-triggered systems are generally more accessible within existing clinical workflows but may require improvements in release efficiency, safety, or formulation scalability to enable broader adoption. No single modality currently fulfills all the desired performance criteria for externally triggered release. Future advances are likely to emerge from integrating multiple triggering mechanisms within a single platform or employing sequential activation strategies to optimize both therapeutic precision and clinical feasibility.

**Table 1:** Summary of triggered drug release characteristics for LBNPs

Triggering stimulus & example LBNPs	LBNP circulation half-life	Release kinetics	Release (%)	Advantages	Disadvantages	References
Thermal (e.g., ThermoDox^®^)	1–2 h	50% in 10–20 min at 42 °C	~ 50%	Well-established mechanism; synergistic with hyperthermia; existing clinical data	Limited thermal control; failed clinical trials; systemic leakiness	[36, 37]
Ultrasound (e.g., Gadoteridol-enhanced, PFP droplets)	2–6 h	1–5 min (low-intensity US), < 1 s (HIFU)	~ 70–90%	Non-invasive; deep tissue penetration; dual imaging and therapy	Mechanism unclear; variable outcomes; no clinical trials yet	[54, 60, 67]
Ionizing Radiation (e.g., GNP-enhanced)	12–48 h	40–60% in 30–60 min	~ 60%	High precision; compatible with existing radiotherapy protocols	Radiation safety concerns; limited LBNP responsiveness	[82]
Magnetic (e.g., nanochain)	~ 26 h	~ 60–80% in 5–10 min	~ 80%	Precise spatial control; long circulation; non-thermal release	Requires specialized RF setup; scalability challenges	[106–108]
Light (e.g., GNP-enhanced, photoactivatable)	12–24 h	< 10 min (laser), 24 h (UV)	~ 70%	High spatial and temporal control; tunable with wavelength	Limited tissue penetration; risk of tissue damage	[87, 111]

## Future perspectives: multi-modal triggered LBNP systems

Emerging research indicates that integrating multiple external stimuli into a single LBNP platform has the potential to enhance the precision, control, and therapeutic efficacy of triggered drug delivery.

### Magneto-light-thermal systems

These LBNPs typically incorporate superparamagnetic IONPs together with NIR photo-absorbers embedded within the LBNP structure, enabling dual heating through AMF-induced magnetic hyperthermia and NIR photothermal conversion [141, 142]. Compared to single-mode systems, this dual approach achieves more efficient heat generation and enables spatiotemporally precise drug release while lowering the required intensity of each stimulus [141]. An additional advantage is their theranostic potential, as the magnetic component can serve as an MRI contrast agent, enabling simultaneous imaging and treatment monitoring [142].

### Magneto-ultrasound systems

Recent reviews have emphasized the promise of magneto-US LBNP systems, in which IONP-loaded liposomes combine magnetic targeting with US-based, real-time, imaging of NPs distribution [143, 144]. Techniques such as magnetomotive US (MMUS) and magneto-acoustic tomography with magnetic induction (MAT-MI) exploit MNP-induced displacement, vibration, or thermoelastic effects to generate US signals that map MNP distribution and guide therapy [143]. Beyond imaging, the integration of US as a secondary stimulus enhances therapeutic control: magneto-US LBNPs achieve improved tumor localization, synergistic bilayer disruption, and more precise drug release compared to single-stimulus systems. A compelling example is the dual-triggered liposome-MB system developed by Dwivedi et al., where Dox-loaded magneto-liposomes (DOX-MLs) were covalently conjugated to PFC MBs (DOX-ML-MBs) [144]. This design enabled magnetic retention combined with US-triggered cavitation, resulting in superior tumor accumulation, deeper NPs penetration into the tumor stroma, and tumor regression with minimal systemic toxicity in vivo [144]. Overall, magneto-US LBNPs provide a multifunctional theranostic platform by (i) enabling magnetic guidance during delivery, (ii) achieving on-demand release via US cavitation or hyperthermia, and (iii) allowing concurrent, non-invasive monitoring of nanoparticle distribution.

### Advantages and challenges of multi-modal triggered LBNP systems

Multi-modal triggered LBNPs offer several advantages over single-stimulus systems, mainly: (i) enhanced release precision through co-localized and complementary triggers; (ii) reduced off-target side-effects by lowering the required intensity of each stimulus; and (iii) theranostic compatibility with imaging modalities and ease of integration into existing diagnostic system [142, 145]. Despite encouraging preclinical data, most multi-modal platforms remain at an early stage of development, much like single-modality triggers. While combining modalities offers clear advantages, these systems still do not fully overcome the limitations inherent to single-mode approaches. A major challenge is the need for rational co-trigger design, that is, selecting stimuli that truly complement each other rather than simply stacking multiple triggers without clear added value. As additional triggers and functionalities are integrated, system complexity inevitably increases, heightening the risk of design-related failures. This underscores the need for not only reliable release mechanisms but also overall stability and structural integrity of the entire LBNP platform. To bridge the gap between laboratory innovation and therapeutic application, future systems must therefore be both clinically adaptable and resilient enough to withstand these added layers of complexity.

## Conclusions

Given the growing interest in precision oncology and the urgent need for safer, more effective drug delivery strategies, this review has critically analyzed recent advances in externally triggered LBNP systems. It provides a timely roadmap for researchers and clinicians aiming to translate these strategies from promising concepts into practical cancer therapies. By examining heat, ultrasound, ionizing radiation, magnetic, and light-based approaches, this review underscores both the unique strengths of each modality and the trade-offs that must be balanced between technical complexity and clinical feasibility, while also offering a rational and quantitative analysis to guide future development. Despite significant progress at the preclinical level, the clinical translation of externally triggered drug release systems remains limited. Major barriers include the physiological complexity and heterogeneity of the TME, immune responses not adequately captured in small animal models, and the technical difficulty of synchronizing rapid, localized drug release with peak nanoparticle circulation times. These challenges demand careful formulation design, rigorous optimization, and comprehensive clinical validation. To achieve meaningful clinical impact, next-generation externally triggered systems should meet four critical benchmarks:

1. Prolonged systemic circulation to ensure steady availability of drug-loaded carriers at the tumor site.
2. Stable payload retention within the LBNPs under physiological conditions, minimizing premature leakage.
3. Stimulus-responsive core design, enabling high drug loading alongside an agent that rapidly disrupts the bilayer upon external activation, achieving drug release ideally within seconds to one minute.
4. Scalable and safe manufacturing, relying on biocompatible, non-toxic materials and processes suitable for regulatory approval.

Meeting these criteria has the potential to yield tumor drug exposures many times greater than conventional chemotherapeutic drugs, potentially redefining the therapeutic ceiling of systemic chemotherapy. Future research should prioritize refining trigger-response mechanisms, leveraging advanced nanofabrication and materials engineering, and integrating these systems into clinically feasible treatment protocols. If these challenges are met, externally triggered LBNPs could transform systemic chemotherapy into a more precise, effective, and less toxic cancer therapy, ultimately improving survival and quality of life for patients.

## Data Availability

No datasets were generated or analysed during the current study.
